# Supplementary material for: Reference-free cell type deconvolution of multi-cellular pixel-resolution spatially resolved transcriptomics data
Source: Nat Commun. 2022 Apr 29;13:2339. doi: 10.1038/s41467-022-30033-z (PMC9055051; doi:10.1038/s41467-022-30033-z)
Supplement: Supplementary file 2 — Reporting Summary [file 41467_2022_30033_MOESM2_ESM.pdf]

nature research

Corresponding author(s): Dr. Jean Fan

Last updated by author(s): Mar 30, 2022

Reporting Summary

Nature Research wishes to improve the reproducibility of the work that we publish. This form provides structure for consistency and transparency in reporting. For further information on Nature Research policies, see our Editorial Policies and the Editorial Policy Checklist.

Statistics

For all statistical analyses, confirm that the following items are present in the figure legend, table legend, main text, or Methods section.

n/a	Confirmed	
[ ]	[x]	The exact sample size (*n*) for each experimental group/condition, given as a discrete number and unit of measurement
[ ]	[x]	A statement on whether measurements were taken from distinct samples or whether the same sample was measured repeatedly
[ ]	[x]	The statistical test(s) used AND whether they are one- or two-sided<br>*Only common tests should be described solely by name; describe more complex techniques in the Methods section.*
[x]	[ ]	A description of all covariates tested
[ ]	[x]	A description of any assumptions or corrections, such as tests of normality and adjustment for multiple comparisons
[ ]	[x]	A full description of the statistical parameters including central tendency (e.g. means) or other basic estimates (e.g. regression coefficient) AND variation (e.g. standard deviation) or associated estimates of uncertainty (e.g. confidence intervals)
[ ]	[x]	For null hypothesis testing, the test statistic (e.g. *F*, *t*, *r*) with confidence intervals, effect sizes, degrees of freedom and *P* value noted<br>*Give P values as exact values whenever suitable.*
[ ]	[x]	For Bayesian analysis, information on the choice of priors and Markov chain Monte Carlo settings
[x]	[ ]	For hierarchical and complex designs, identification of the appropriate level for tests and full reporting of outcomes
[ ]	[x]	Estimates of effect sizes (e.g. Cohen's *d*, Pearson's *r*), indicating how they were calculated

Our web collection on statistics for biologists contains articles on many of the points above.

Software and code

Policy information about availability of computer code

Data collection	No software was used.
Data analysis	- The STdeconvolve software package version 0.1.0 was used to analyze the data. The source code is publicly available at https://github.com/JEFworks-Lab/STdeconvolve.<br>- Rank-based gene set enrichment analysis was performed using `liger` version 2.0.1.<br>- Runtime and memory testing were performed using `microbenchmark` version 1.4.7 and `profmem` version 0.6.0.<br>- Supervised deconvolution was performed using methods `SPOTlight` version 0.1.7, `RCTD` version 1.2.0, and `spatialDWLS` implemented through `Giotto` version 2.0.0.953.

For manuscripts utilizing custom algorithms or software that are central to the research but not yet described in published literature, software must be made available to editors and reviewers. We strongly encourage code deposition in a community repository (e.g. GitHub). See the Nature Research guidelines for submitting code & software for further information.

Data

Policy information about availability of data

All manuscripts must include a data availability statement. This statement should provide the following information, where applicable:
- Accession codes, unique identifiers, or web links for publicly available datasets
- A list of figures that have associated raw data
- A description of any restrictions on data availability

MERFISH mouse medial preoptic area (Moffit et al. 2018).
Cell centroid coordinates, gene counts, and metadata are available for download at https://datadryad.org/stash/dataset/doi:10.5061/dryad.8t8s248/.

MERFISH mouse coronal section of the cortex.
Cell centroid coordinates, gene counts, and metadata of Slice 2, replicate 1 available as part of the Vizgen Data Release V1.0. May 2021. https://info.vizgen.com/mouse-brain-data.

Mouse olfactory bulb (MOB) (Stahl et al. 2016).
Gene count matrices and H&E images for all MOB replicates are available for download at https://www.spatialresearch.org/resources-published-datasets/doi-10-1126science-aaf2403/.

Breast cancer sections (BCL) (Yoosuf et al. 2020).
Gene count matrices and H&E images for all BCL replicates are available for download at https://www.spatialresearch.org/resources-published-datasets/doi-10-1126science-aaf2403/.

10X Visium data of the mouse coronal section of the cortex (V1_Adult_Mouse_Brain - Adult Mouse Brain (Coronal)) available for download at https://www.10xgenomics.com/resources/datasets/mouse-brain-section-coronal-1-standard-1-1-0.

DBiT-seq dataset of E11 mouse embryo lower body (GSM4364242_E11-1L). Gene count matrices available for download at https://www.ncbi.nlm.nih.gov/geo/query/acc.cgi?acc=GSE137986.

Slide-Seq of the mouse cerebellum (Puck_180819_12) available for download at https://singlecell.broadinstitute.org/single_cell/study/SCP354/slide-seq-study#study-download.

scRNA-seq data for simulations (Li et al. 2020)
Single-cell gene count matrices and cell metadata available for download at https://www.ncbi.nlm.nih.gov/geo/query/acc.cgi?acc=GSE150580.

scRNA-seq data of the mouse olfactory bulb (Tepe et al. 2018) available for download at https://www.ncbi.nlm.nih.gov/geo/query/acc.cgi?acc=GSE121891.

Drop-seq single-cell RNA-seq of mouse cerebellum available for download at http://dropviz.org/.

Field-specific reporting

Please select the one below that is the best fit for your research. If you are not sure, read the appropriate sections before making your selection.

- [x] Life sciences
- [ ] Behavioural & social sciences
- [ ] Ecological, evolutionary & environmental sciences

For a reference copy of the document with all sections, see nature.com/documents/nr-reporting-summary-flat.pdf

Life sciences study design

All studies must disclose on these points even when the disclosure is negative.

Sample size	For the mouse medial preoptic area MERFISH data all 12 tissue sections were used in the analysis. Simulating spatial transcriptomics pixels from this data at 100um, 50um, 20um, and 10um resolutions yielded 3072, 13477, 45978, and 57397 total pixels respectively. For the mouse olfactory bulb ST data, replicates 8, 2, 5, and 12 were used. The number of pixels contained within these datasets after filtering out poor pixels was 260, 279, 267, and 278, respectively. For the breast cancer data ST data, all 4 sections were used resulting in a total of 1029 pixels after filtering. For the Slide-Seq of the mouse cerebellum, Puck_180819_12 was used. For the DBiT-seq data of E11 mouse embryo lower body, sample GSM4364242_E11-1L was used, which contained 1831 pixels after filtering. For 10X Visium, the V1_Adult_Mouse_Brain with v1 Chemistry was used.
Data exclusions	For the MERFISH data, we excluded any cells previously annotated as "Ambiguous". For the MOB ST datasets, we excluded pixels with less than 100 total gene counts. For the breast cancer ST data, we excluded pixels with less than 10 total gene counts. For the Slide-seq data, we excluded beads with less than 50 total gene counts. For DBiT-seq, we excluded pixels with less than 100 total gene counts. For 10X Visium data, we excluded pixels with less than 100 total gene counts.
Replication	For the MOB ST datasets, findings are replicated across all datasets used. For the breast cancer ST datasets, findings are replicated across sections. For assessing the accuracy of STdeconvolve with respect to dataset size, we repeated our analysis 10 times for every subsampling of pixels. For assessing the accuracy of STdeconvolve with respect to the number of feature selected genes, we repeated our analysis 3 times for every subsampling of genes. All other datasets were analyzed once.
Randomization	Not relevant because each dataset analyzed separately and comparisons only involve groups of pixels within a given dataset.
Blinding	STdeconvolve was applied to deconvolve the dataset pixels in an unsupervised way. After, the model predictions were compared to previous annotations or ground truths. Thus, blinding was not relevant to this study.

Reporting for specific materials, systems and methods

We require information from authors about some types of materials, experimental systems and methods used in many studies. Here, indicate whether each material, system or method listed is relevant to your study. If you are not sure if a list item applies to your research, read the appropriate section before selecting a response.

Materials & experimental systems

n/a	Involved in the study
☒	☐ Antibodies
☒	☐ Eukaryotic cell lines
☒	☐ Palaeontology and archaeology
☒	☐ Animals and other organisms
☒	☐ Human research participants
☒	☐ Clinical data
☒	☐ Dual use research of concern

Methods

n/a	Involved in the study
☒	☐ ChIP-seq
☒	☐ Flow cytometry
☒	☐ MRI-based neuroimaging
