# Supplementary material for: Reference-free cell type deconvolution of multi-cellular pixel-resolution spatially resolved transcriptomics data
Source: Nat Commun. 2022 Apr 29;13:2339. doi: 10.1038/s41467-022-30033-z (PMC9055051; doi:10.1038/s41467-022-30033-z)
Supplement: Supplementary file 4 — Description of Additional Supplementary Files [file 41467_2022_30033_MOESM4_ESM.pdf]

Title: Supplementary Data 1.

Description: Significant GO terms for STdeconvolve cell-type X15 derived from the breast cancer ST dataset. Table columns: “term” = GO term; “p.val” = p-value; “q.val” = multiple testing adjusted p-value; “sscore” = GO term enrichment score; “edge” = GO term edge score. Significance was determined by permutation testing during gene set enrichment analysis[1]. Multiple testing correction was performed using the Benjamini and Hochberg method[2].

1 Subramanian, A. et al. Gene set enrichment analysis: a knowledge-based approach for interpreting genome-wide expression profiles. Proc Natl Acad Sci U S A 102, 15545- 15550, doi:10.1073/pnas.0506580102 (2005).

2 Benjamini, Y. & Hochberg, Y. Controlling the False Discovery Rate: A Practical and Powerful Approach to Multiple Testing. Journal of the Royal Statistical Society. Series B (Methodological) 57, 289-300 (1995).
