# Supplementary material for: Reference-free cell type deconvolution of multi-cellular pixel-resolution spatially resolved transcriptomics data
Source: Nat Commun. 2022 Apr 29;13:2339. doi: 10.1038/s41467-022-30033-z (PMC9055051; doi:10.1038/s41467-022-30033-z)
Supplement: Supplementary file 6 — Supplementary Software [file 41467_2022_30033_MOESM6_ESM.gz › STdeconvolve/inst/doc/vignette.html]

STdeconvolve Vignette


# STdeconvolve Vignette

Brendan F. Miller

#### 30 March 2022

# Contents

- 1 Installation
- 2 Introduction
  - 2.1 Deconvolution
  - 2.2 Visualization
  - 2.3 Annotation Strategy 1: Transcriptional correlations
  - 2.4 Annotation Strategy 2: GSEA
- 3 `SpatialExperiment` inputs
- 4 Additional tutorials and analyses
- 5 Session information

# 1 Installation

```
if(!requireNamespace("BiocManager", quietly = TRUE))
    install.packages("BiocManager")
BiocManager::install("STdeconvolve")
```

# 2 Introduction

`STdeconvolve` is an unsupervised machine learning approach to deconvolve
multi-cellular pixel-resolution spatial transcriptomics datasets in order to
recover the putative transcriptomic profiles of cell-types and their
proportional representation within spatially resolved pixels without reliance on
external single-cell transcriptomics references.

## 2.1 Deconvolution

In this tutorial, we will walk through some of the main functionalities of
`STdeconvolve`.

```
library(STdeconvolve)
```

Given a counts matrix from pixel-resolution spatial transcriptomics data where
each spatially resolved measurement may represent mixtures from potentially
multiple cell-types, `STdeconvolve` infers the putative transcriptomic profiles of
cell-types and their proportional representation within each multi-cellular
spatially resolved pixel. Such a pixel-resolution spatial transcriptomics
dataset of the mouse olfactory bulb is built in and can be loaded.

```
data(mOB)
pos <- mOB$pos ## x and y positions of each pixel
cd <- mOB$counts ## matrix of gene counts in each pixel
annot <- mOB$annot ## annotated tissue layers assigned to each pixel
```

`STdeconvolve` first feature selects for genes most likely to be relevant for
distinguishing between cell-types by looking for highly overdispersed genes
across ST pixels. Pixels with too few genes or genes with too few reads can also
be removed.

```
## remove pixels with too few genes
counts <- cleanCounts(counts = cd,
                      min.lib.size = 100,
                      min.reads = 1,
                      min.detected = 1,
                      verbose = TRUE)
```

```
## Converting to sparse matrix ...
```

```
## Filtering matrix with 262 cells and 15928 genes ...
```

```
## Resulting matrix has 260 cells and 14828 genes
```

```
## feature select for genes
corpus <- restrictCorpus(counts,
                         removeAbove = 1.0,
                         removeBelow = 0.05,
                         alpha = 0.05,
                         plot = TRUE,
                         verbose = TRUE)
```

```
## Removing 124 genes present in 100% or more of pixels...
```

```
## 14704 genes remaining...
```

```
## Removing 3009 genes present in 5% or less of pixels...
```

```
## 11695 genes remaining...
```

```
## Restricting to overdispersed genes with alpha = 0.05...
```

```
## Calculating variance fit ...
```

```
## Using gam with k=5...
```

```
## 232 overdispersed genes ...
```

```
##  Using top 1000 overdispersed genes.
```

```
##  number of top overdispersed genes available: 232
```

`STdeconvolve` then applies latent Dirichlet allocation (LDA), a generative
statistical model commonly used in natural language processing, to discover `K`
latent cell-types. `STdeconvolve` fits a range of LDA models to inform the
choice of an optimal `K`.

```
## Note: the input corpus needs to be an integer count matrix of pixels x genes
ldas <- fitLDA(t(as.matrix(corpus)), Ks = seq(2, 9, by = 1),
               perc.rare.thresh = 0.05,
               plot=TRUE,
               verbose=TRUE)
```

```
## Time to fit LDA models was 1.13 mins
```

```
## Computing perplexity for each fitted model...
```

```
## Time to compute perplexities was 0 mins
```

```
## Getting predicted cell-types at low proportions...
```

```
## Time to compute cell-types at low proportions was 0 mins
```

```
## Plotting...
```

In this example, we will use the model with the lowest model perplexity.

The shaded region indicates where a fitted model for a given K had an
`alpha` > 1. `alpha` is an LDA parameter that is solved for during model
fitting and corresponds to the shape parameter of a symmetric Dirichlet
distribution. In the model, this Dirichlet distribution describes the cell-type
proportions in the pixels. A symmetric Dirichlet with `alpha` > 1 would lead to
more uniform cell-type distributions in the pixels and difficulty identifying
distinct cell-types. Instead, we want models with `alpha` < 1, resulting in
sparse distributions where only a few cell-types are represented in a given
pixel.

The resulting `theta` matrix can be interpreted as the proportion of each
deconvolved cell-type across each spatially resolved pixel. The resulting `beta`
matrix can be interpreted as the putative gene expression profile for each
deconvolved cell-type normalized to a library size of 1. This `beta` matrix can
be scaled by a depth factor (ex. 1000) for interpretability.

```
## select model with minimum perplexity
optLDA <- optimalModel(models = ldas, opt = "min")

## Extract pixel cell-type proportions (theta) and cell-type gene expression
## profiles (beta) for the given dataset.
## We can also remove cell-types from pixels that contribute less than 5% of the
## pixel proportion and scale the deconvolved transcriptional profiles by 1000 
results <- getBetaTheta(optLDA,
                        perc.filt = 0.05,
                        betaScale = 1000)
```

```
## Filtering out cell-types in pixels that contribute less than 0.05 of the pixel proportion.
```

```
deconProp <- results$theta
deconGexp <- results$beta
```

## 2.2 Visualization

We can now visualize the proportion of each deconvolved cell-type across the
original spatially resolved pixels.

```
vizAllTopics(deconProp, pos, 
             groups = annot, 
             group_cols = rainbow(length(levels(annot))),
             r=0.4)
```

```
## Plotting scatterpies for 260 pixels with 8 cell-types...this could take a while if the dataset is large.
```

For faster plotting, we can visualize the pixel proportions of a single
cell-type separately using `vizTopic()`:

```
vizTopic(theta = deconProp, pos = pos, topic = "5", plotTitle = "X5",
         size = 5, stroke = 1, alpha = 0.5,
         low = "white",
         high = "red")
```

With deconvolved cell-types in hand, we will now go over two different
strategies for annotating the deconvolved cell-types.

Recall that `STdeconvolve` does not require a reference to deconvolve cell-types
in multi-cellular spatially-resolved pixels. However, we would still like some
way to identify the deconvolved cell-types to determine if they may represent
known cell-types.

In addition to the predicted pixel proportions, `STdeconvolve` also returns
predicted transcriptional profiles of the deconvolved cell-types as the `beta`
matrix. We can use these transcriptional profiles to compare to known cell-type
transcriptional profiles and see if we can annotate them.

For demonstration purposes, let’s use the 5 annotated tissue layer labels
(i.e. “Granular Cell Layer”, “Mitral Cell Layer”, etc) assigned to each pixel
and use these to make transcriptional profiles for each of the annotated tissue
layers in the MOB.

```
# proxy theta for the annotated layers
mobProxyTheta <- model.matrix(~ 0 + annot)
rownames(mobProxyTheta) <- names(annot)
# fix names
colnames(mobProxyTheta) <- unlist(lapply(colnames(mobProxyTheta), function(x) {
  unlist(strsplit(x, "annot"))[2]
}))

mobProxyGexp <- counts %*% mobProxyTheta
```

## 2.3 Annotation Strategy 1: Transcriptional correlations

First, we can find the Pearson’s correlation between the transcriptional
profiles of the deconvolved cell-types and those of a ground truth reference.

```
corMtx_beta <- getCorrMtx(# the deconvolved cell-type `beta` (celltypes x genes)
                          m1 = as.matrix(deconGexp),
                          # the reference `beta` (celltypes x genes)
                          m2 = t(as.matrix(mobProxyGexp)),
                          # "b" = comparing beta matrices, "t" for thetas
                          type = "b")
```

```
## NOTE: using type='b' and comparing betas where the cell-types are
##             the rows (cell-types x genes)
```

```
## cell-type correlations based on 232 shared genes between m1 and m2.
```

```
## row and column names need to be characters
rownames(corMtx_beta) <- paste0("decon_", seq(nrow(corMtx_beta)))

correlationPlot(mat = corMtx_beta,
                # colLabs (aka x-axis, and rows of matrix)
                colLabs = "Deconvolved cell-types",
                # rowLabs (aka y-axis, and columns of matrix)
                rowLabs = "Ground truth cell-types",
                title = "Transcriptional correlation", annotation = TRUE) +
  ## this function returns a `ggplot2` object, so can add additional aesthetics
  ggplot2::theme(axis.text.x = ggplot2::element_text(angle = 90, vjust = 0))
```

Notice that cell-type 1, 4, and 5 correlate the strongest with the Granular cell
layer, cell-type 2 with the Olfactory nerve layer, etc. These agree with there
predicted spatial proportions in the MOB dataset. We can also confirm this by
also computing the correlation between the predicted and ground truth cell-type
proportions via comparing the `theta` matrices.

```
corMtx_theta <- getCorrMtx(# deconvolved cell-type `theta` (pixels x celltypes)
                           m1 = as.matrix(deconProp),
                           # the reference `theta` (pixels x celltypes)
                           m2 = as.matrix(mobProxyTheta),
                           # "b" = comparing beta matrices, "t" for thetas
                           type = "t")
```

```
## NOTE: using type='t' and comparing thetas where the cell-types are
##             the columns (pixels x cell-types)
```

```
## cell-type correlations based on 260 shared pixels between m1 and m2.
```

```
## row and column names need to be characters
rownames(corMtx_theta) <- paste0("decon_", seq(nrow(corMtx_theta)))

correlationPlot(mat = corMtx_theta,
                # colLabs (aka x-axis, and rows of matrix)
                colLabs = "Deconvolved cell-types",
                # rowLabs (aka y-axis, and columns of matrix)
                rowLabs = "Ground truth cell-types",
                title = "Proportional correlation", annotation = TRUE) +
  ## this function returns a `ggplot2` object, so can add additional aesthetics
  ggplot2::theme(axis.text.x = ggplot2::element_text(angle = 90, vjust = 0))
```

Finally, we can also pair up each reference cell-type with the deconvolved
cell-type that has the highest correlation.

```
## Order the cell-types rows based on best match (highest correlation) with
## each community.
## Cannot have more rows than columns for this pairing, so transpose
pairs <- lsatPairs(t(corMtx_theta))
m <- t(corMtx_theta)[pairs$rowix, pairs$colsix]

correlationPlot(mat = t(m), # transpose back
                # colLabs (aka x-axis, and rows of matrix)
                colLabs = "Deconvolved cell-types",
                # rowLabs (aka y-axis, and columns of matrix)
                rowLabs = "Ground truth cell-types",
                title = "Transcriptional correlation", annotation = TRUE) +
  ## this function returns a `ggplot2` object, so can add additional aesthetics
  ggplot2::theme(axis.text.x = ggplot2::element_text(angle = 90, vjust = 0))
```

Note that only the paired deconvolved cell-types remain. Ones that paired less
strongly with a given ground truth are dropped after assigning pairs.

## 2.4 Annotation Strategy 2: GSEA

Next, given a list of reference gene sets for different cell types, we can
performed gene set enrichment analysis on the deconvolved transcriptional
profiles to test for significant enrichment of any known ground truth cell-types.

First, let’s identify marker genes for each tissue layer based on
log2(fold-change) compared to the other tissue layers. This will be our list of
gene sets for each tissue layer.

```
mobProxyLayerMarkers <- list()

## make the tissue layers the rows and genes the columns
gexp <- t(as.matrix(mobProxyGexp))

for (i in seq(length(rownames(gexp)))){
  celltype <- i
  ## log2FC relative to other cell-types
  ## highly expressed in cell-type of interest
  highgexp <- names(which(gexp[celltype,] > 10))
  ## high log2(fold-change) compared to other deconvolved cell-types and limit
  ## to the top 200
  log2fc <- sort(
                log2(gexp[celltype,highgexp]/colMeans(gexp[-celltype,highgexp])),
                decreasing=TRUE)[1:200]
  
  ## for gene set of the ground truth cell-type, get the genes
  ## with log2FC > 1 (so FC > 2 over the mean exp of the other cell-types)
  markers <- names(log2fc[log2fc > 1])
  mobProxyLayerMarkers[[ rownames(gexp)[celltype] ]] <- markers
}
```

```
celltype_annotations <- annotateCellTypesGSEA(beta = results$beta,
                                              gset = mobProxyLayerMarkers,
                                              qval = 0.05)
```

`annotateCellTypesGSEA` returns a list where the first entry, `$results`,
contains a list of matrices that show any reference cell-types that had a
significant positive enrichment score in each of the deconvolved cell-types.

For example, here are the reference cell-types that were significantly enriched
in deconvolved cell-type 2:

```
celltype_annotations$results$`2`
```

Note that the “5: Olfactory Nerve Layer” is significantly positively enriched in
the transcriptional profiles of cell-type 2 whereas “1: Granular Cell Layer” is
negatively enriched.

`annotateCellTypesGSEA` also contains `$predictions`, which is a named vector of
the most significant matched reference cell-type with the highest positive
enrichment score for each deconvolved cell-type. Note that if there were no
significant reference cell-types with positive enrichment, then the deconvolved
cell-type will have no matches.

```
celltype_annotations$predictions
```

Note how the best matches are closely associated with the transcriptional and
pixel proportion correlations.

# 3 `SpatialExperiment` inputs

Spatial datasets stored as a (`SpatialExperiment`)[https://bioconductor.org/packages/release/bioc/html/SpatialExperiment.html]
object, and as an extension, 10X Visium datasets made available through (`TENxVisiumData`)[https://bioconductor.org/packages/release/data/experiment/html/TENxVisiumData.html],
can be accessed the following way:

```
## install `SpatialExperiment` and `TENxVisiumData` if not already
if (!require("BiocManager", quietly = TRUE))
    install.packages("BiocManager")
BiocManager::install(c("SpatialExperiment", "TENxVisiumData"))
```

```
library(SpatialExperiment)
library(TENxVisiumData)

## load the MouseBrainCoronal SpatialExperiment object from `TENxVisiumData`
se <- TENxVisiumData::MouseBrainCoronal()
```

Alternatively one can also create a `SpatialExperiment` object from the
downloaded Visium mouse brain section (coronal) dataset
directly. In particular, we are interested in the filtered count matrix and the
spatial positions of the barcodes.

First, make a directory to store the downloaded files:

```
f <- "visiumTutorial/"

if(!file.exists(f)){
      dir.create(f)
  }
```

Download and unzip the `Feature / barcode matrix (filtered)` and the
`Spatial imaging data`:

```
if(!file.exists(paste0(f, "V1_Adult_Mouse_Brain_filtered_feature_bc_matrix.tar.gz"))){
  tar_gz_file <- "http://cf.10xgenomics.com/samples/spatial-exp/1.1.0/V1_Adult_Mouse_Brain/V1_Adult_Mouse_Brain_filtered_feature_bc_matrix.tar.gz"
  download.file(tar_gz_file, 
                destfile = paste0(f, "V1_Adult_Mouse_Brain_filtered_feature_bc_matrix.tar.gz"), 
                method = "auto")
}
untar(tarfile = paste0(f, "V1_Adult_Mouse_Brain_filtered_feature_bc_matrix.tar.gz"), 
      exdir = f)

if(!file.exists(paste0(f, "V1_Adult_Mouse_Brain_spatial.tar.gz"))){
spatial_imaging_data <- "http://cf.10xgenomics.com/samples/spatial-exp/1.1.0/V1_Adult_Mouse_Brain/V1_Adult_Mouse_Brain_spatial.tar.gz"
  download.file(spatial_imaging_data, 
                destfile = paste0(f, "V1_Adult_Mouse_Brain_spatial.tar.gz"), 
                method = "auto")
}
untar(tarfile = paste0(f, "V1_Adult_Mouse_Brain_spatial.tar.gz"), 
      exdir = f)
```

Load the filtered counts and spatial barcode information into a
`SpatialExperiment`:

```
se <- SpatialExperiment::read10xVisium(samples = f,
     type = "sparse",
     data = "filtered")
```

From here, the count matrix can be accessed and setup for feature selection in
`STdeconvolve` via:

```
## this is the genes x barcode sparse count matrix
cd <- se@assays@data@listData$counts
```

“x” and “y” coordinates of the barcodes can be obtained via:

```
pos <- SpatialExperiment::spatialCoords(se)

## change column names to x and y
## for this dataset, we will visualize barcodes using
## "pxl_col_in_fullres" = "y" coordinates,
## and "pxl_row_in_fullres" = "x" coordinates
colnames(pos) <- c("y", "x")
```

Poor genes and barcodes will be removed from the count matrix

```
counts <- cleanCounts(cd, min.lib.size = 100, min.reads = 10)
```

And then we can feature select for overdispersed genes that are present in less
than 100% of the barcodes and more than 5%.

We will also use the top 1000 most significant overdispersed genes by default.

```
corpus <- restrictCorpus(counts,
                         removeAbove=1.0,
                         removeBelow = 0.05,
                         nTopOD = 1000)
```

Now, we can fit an LDA model to the feature selected corpus, with K=15

```
ldas <- fitLDA(t(as.matrix(corpus)), Ks = c(15))
```

Next, select the LDA model of interest and get the **beta** (cell-type
transcriptional profiles) and **theta** (cell-type barcode proportions) matrices.

```
optLDA <- optimalModel(models = ldas, opt = 15)

results <- getBetaTheta(optLDA, perc.filt = 0.05, betaScale = 1000)
deconProp <- results$theta
deconGexp <- results$beta
```

Now, we can visualize the deconvolved cell-type proprotions in each spot.

```
plt <- vizAllTopics(theta = deconProp,
                   pos = pos,
                   r = 45,
                   lwd = 0,
                   showLegend = TRUE,
                   plotTitle = NA) +
  ggplot2::guides(fill=ggplot2::guide_legend(ncol=2)) +
  
  ## outer border
  ggplot2::geom_rect(data = data.frame(pos),
            ggplot2::aes(xmin = min(x)-90, xmax = max(x)+90,
                         ymin = min(y)-90, ymax = max(y)+90),
            fill = NA, color = "black", linetype = "solid", size = 0.5) +
  
  ggplot2::theme(
    plot.background = ggplot2::element_blank()
  ) +
  
  ## remove the pixel "groups", which are color aesthetics for the pixel borders
  ggplot2::guides(colour = "none")
```

# 4 Additional tutorials and analyses

For additional tutorials and commands to reproduce the preprocessing of certain
datasets used in the manuscript, check out:

https://jef.works/STdeconvolve/

# 5 Session information

```
sessionInfo()
```
